# Progressive necrotic mass of the midline face

**DOI:** 10.1016/j.jdcr.2024.06.024

**Published:** 2024-07-24

**Authors:** Clark Gravell, Ndanzia Mpunga, Alison Messer, Adolfo Diaz Duque

**Affiliations:** a Department of Dermatology, UT Health San Antonio, San Antonio, Texas; b UT Health San Antonio, Joe R. & Teresa Lozano Long School of Medicine, San Antonio, Texas; c Department of Hematology, UT Health San Antonio, San Antonio, Texas

**Keywords:** Epstein-Barr virus, extranodal NK/T cell lymphoma, midline face, nasal, necrotic

## History of present illness

**Fig 1:**
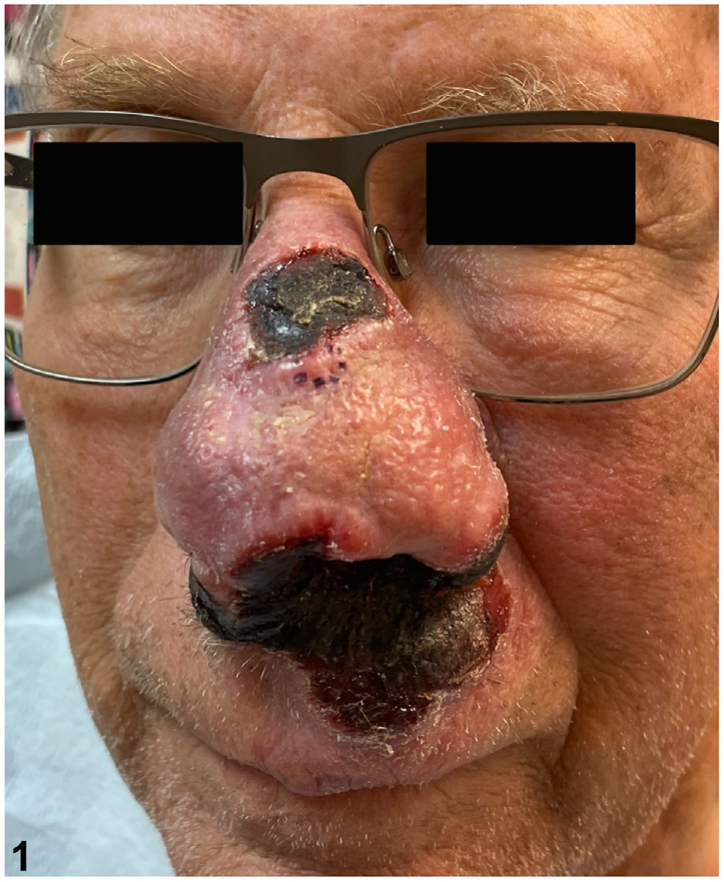

**Fig 2:**
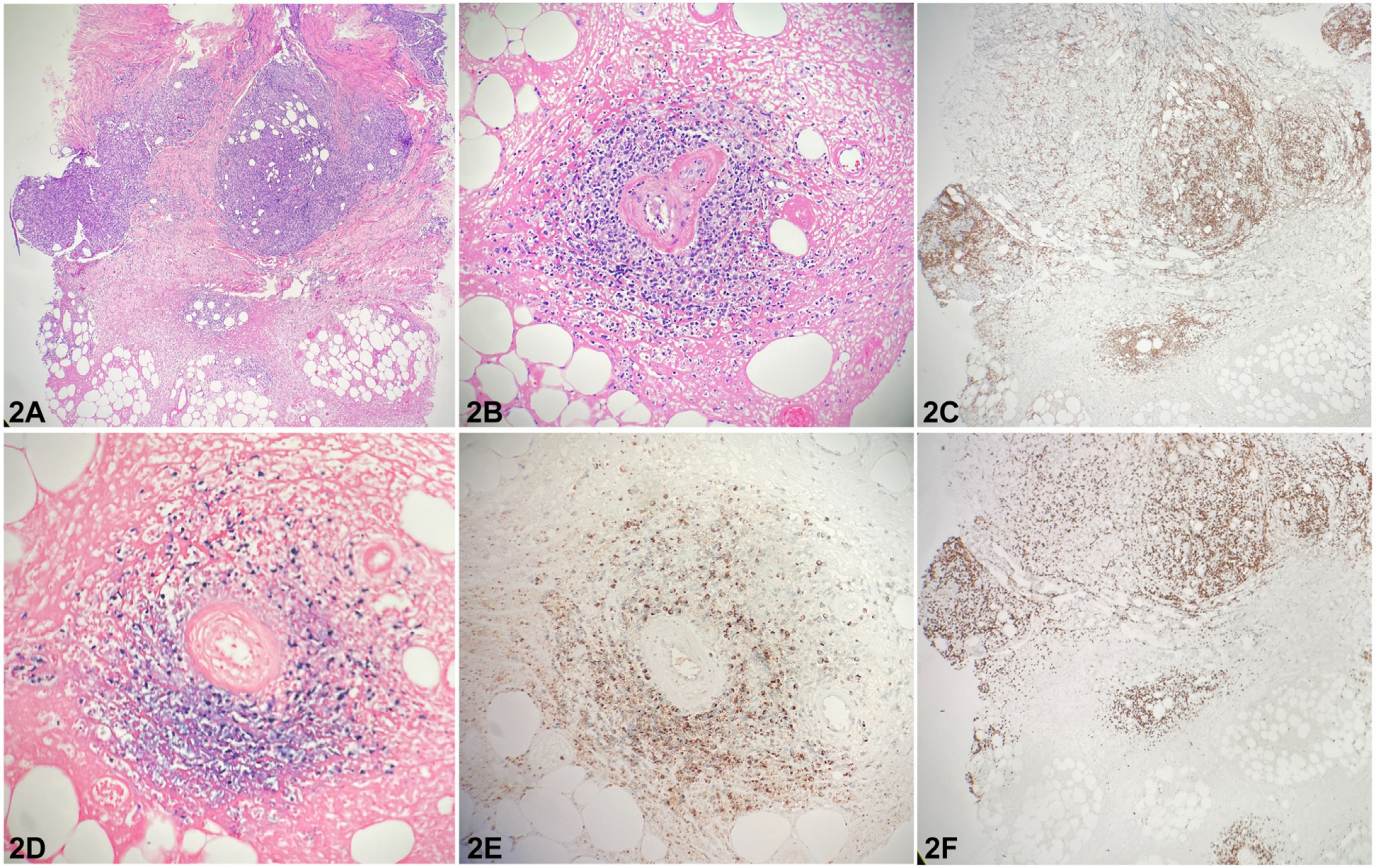

A 74-year-old man with no pertinent dermatologic history presented to the clinic for nasal mass. The changes to the nose began 3 months prior, initially appearing as non-tender, mildly pruritic nasal swelling, and erythema. Physical examination revealed a violaceous tumor of the nose and upper cutaneous lip with multiple areas of eschar formation (Fig 1). Histopathological examination of the lesion (Fig 2) demonstrated dense nodular aggregates of atypical cells on hematoxylin and eosin stain (2a, 2b) that stained positive for CD56 (2c), Epstein-Barr virus (EBV)-encoded small RNA (EBER) (2d), and T-cell intracellular antigen 1 (2e) with high proliferation index as indicated by Ki67 (2f).

**Question 1: What is the most likely diagnosis?**

A. Granulomatosis with polyangiitis
B. Extranodal NK/T-cell lymphoma (ENKTCL)
C. Mucormycosis
D. Leishmaniasis
E. Herpes simplex virus 1 (HSV-1) infection

**Answers:**

A. Granulomatosis with polyangiitis – Incorrect. Although granulomatosis with polyangiitis most often presents in the upper airway and can manifest as ulceration of the nose,^1^ pathology would show leukocytoclastic vasculitis involving small-sized vessels.
B. Extranodal NK/T-cell lymphoma (ENKTCL) – Correct. ENKTCL is a rapidly progressive form of non-Hodgkin’s lymphoma that can present with extensive midline necrosis of the face which may resemble conditions such as cellulitis, mucormycosis, or granulomatosis with polyangiitis.^1^ Microscopic evaluation will typically demonstrate pleomorphic lymphoid cells in an angiocentric pattern expressing a combination of CD3, CD56, T-cell intracellular antigen 1, and EBER.^1^, ^2^, ^3^
C. Mucormycosis – Incorrect. Mucormycosis is a rare, angioinvasive fungal infection that typically occurs in diabetic patients with poor glycemic control. Pathology commonly shows broad, hollow-appearing hyphae in the setting of tissue necrosis.
D. Leishmaniasis – Incorrect. Leishmaniasis is a protozoal infection caused by *Leishmania spp.* which may present as a progressive ulcerative papule on the nose with possible extension to the nasal septum, lips, and palate. Histopathology will typically reveal a granulomatous dermatitis with parasitized dermal macrophages with cytoplasmic clusters of *Leishmania* amastigotes randomly spaced or lined up at the periphery of the vacuole said to resemble a movie marquee.
E. Herpes simplex virus 1 (HSV-1) infection – Incorrect. HSV-1 classically presents as a cluster of vesicles on an erythematous base. Although immunocompromised patients may present with atypical findings such as necrotic ulcers or exophytic masses, histopathology often shows ballooning degeneration of keratinocytes and multinucleated keratinocytes with nuclear molding that stains positive for HSV antigen.

**Question 2: Which of the following is the best next step in management?**

A. Admit to hospital for surgical debridement and intravenous antifungals
B. Admit to hospital for incision and drainage with intravenous antibiotics
C. Prescribe oral valacyclovir and follow-up in 1 month
D. Obtain positron emission tomography-computed tomography (PET/CT) to confirm tumor stage and extent of disease
E. Start outpatient chemotherapy and radiation with weekly follow-up

**Answers:**

A. Admit to hospital for surgical debridement and intravenous antifungals – Incorrect. ENKTCL is commonly misdiagnosed as fungal infections such as mucormycosis, leading to delayed treatment.^1^ Patients with ENKTCL will not show improvement of skin lesions with antifungals, which should warrant repeat biopsy.
B. Admit to hospital for incision and drainage with intravenous antibiotics – Incorrect. ENKTCL can be misdiagnosed for deep tissue bacterial infections such as facial cellulitis, which may lead to unnecessary antibiotic treatment and surgical procedures without symptom improvement.^1^
C. Prescribe oral valacyclovir and follow-up in 1 month – Incorrect. Valacyclovir would be the appropriate medication for HSV infection, which may exhibit an atypical presentation that mimics ENKTCL. However, oral antivirals will not be effective in this situation and may delay proper treatment of ENKTCL.
D. Obtain positron emission tomography-computed tomography (PET/CT) to confirm tumor stage and extent of disease – Correct. Patients diagnosed with ENKTCL often present with extranodal involvement at the time of diagnosis, therefore the National Cancer Comprehensive Network recommends the use of PET/CT scans for staging to further guide management.^4^ Our patient’s PET/CT showed a hypermetabolic mass affecting the nose and hypermetabolic activity on the distal right leg suggestive of extranodal involvement, so he was started on gemcitabine, dexamethasone, and cisplatin chemotherapy with plans for sandwiched radiotherapy. To date, patient is status post-five cycles of chemotherapy with significant clinical improvement in nasal mass and overall decreased metabolic response on PET/CT.
E. Start outpatient chemotherapy and radiation with weekly follow-up – Incorrect. Although patients with ENKTCL are most often treated with chemotherapy and/or radiation therapy, beginning chemotherapy immediately is premature. Staging should be performed first to assess for metastatic disease, which affects induction therapy.^3^^,^^4^

**Question 3: The pathogenesis of ENKTCL is thought to have a significant association with past infection by which of the following organisms?**

A. EBV
B. Human papillomavirus (HPV)
C. Polyomavirus
D. Human herpesvirus 8 (HHV-8)
E. Varicella zoster virus (VZV)

**Answers:**

A. EBV – Correct. Infection with EBV has been associated with ENKTCL as it is proposed that EBV infection leads to malignant transformation of natural killer T-cells or cytotoxic T-cells.^1^, ^2^, ^3^, ^4^ Detection of EBER with in-situ hybridization is specific for the diagnosis of ENKTCL and plasma EBV viral load is an important factor for prognostic indices of ENKTCL and may serve as a biomarker for treatment response.^2^
B. Human papillomavirus (HPV) – Incorrect. Infection with HPV is associated with an increased risk of anogenital, cervical, and oropharyngeal squamous cell carcinomas.^5^
C. Polyomavirus – Incorrect. Merkel cell polyomavirus, a type of polyoma virus, has been shown to be associated with the development of Merkel cell carcinoma.^5^ Merkel cell carcinoma most commonly presents as a rapidly enlarging pink/purple nodule with ulceration in the sun-exposed areas of the skin.
D. Human herpesvirus 8 (HHV-8) – Incorrect. HHV-8 has been shown to be associated with Kaposi sarcoma, an AIDs-defining illness.^5^ Kaposi sarcoma lesions typically appear as painless, violaceous nodules or plaques most commonly in the face or extremities.
E. Varicella zoster virus (VZV) – Incorrect. Following initial infection with VZV, the virus remains dormant within sensory ganglia of the body. Reactivation most commonly occurs in immunocompromised patients, manifesting with an extremely painful vesiculopapular rash in a dermatomal pattern.

## Conflicts of interest

Dr Diaz Duque is a researcher for Epyzyme; a consultant for Genentech and Lilly; and on the speaker bureau for ADCT, Lilly, AstraZeneca, and Genentech. Dr Gravell, Author Mpunga, and Dr Messer have no conflicts of interest to declare.
